# Metabarcoding analysis provides insight into the link between prey and plant intake in a large alpine cat carnivore, the snow leopard

**DOI:** 10.1098/rsos.240132

**Published:** 2024-05-29

**Authors:** Hiroto Yoshimura, Takashi Hayakawa, Dale M. Kikuchi, Kubanychbek Zhumabai Uulu, Huiyuan Qi, Taro Sugimoto, Koustubh Sharma, Kodzue Kinoshita

**Affiliations:** ^1^ Wildlife Research Center, Kyoto University, Kyoto, Japan; ^2^ Faculty of Environmental Earth Science, Hokkaido University, Sapporo, Hokkaido, Japan; ^3^ Japan Monkey Center, Inuyama, Aichi, Japan; ^4^ Department of Bioresource Development, Tokyo University of Agriculture, Kanagawa, Japan; ^5^ Snow Leopard Foundation in Kyrgyzstan, Bishkek, Kyrgyzstan; ^6^ Institute of Natural and Environmental Sciences, University of Hyogo, Tamba, Hyogo, Japan; ^7^ Snow Leopard Trust, Seattle, WA, USA; ^8^ Graduate School of Asian and African Area Studies, Kyoto University, Kyoto, Japan

**Keywords:** diet analysis, DNA barcoding, carnivore, felids

## Abstract

Species of the family Felidae are thought to be obligate carnivores. However, detection of plants in their faeces raises questions about the role of plants in their diet. This is particularly true for the snow leopard (*Panthera uncia*). Our study aimed to comprehensively identify the prey and plants consumed by snow leopards. We applied DNA metabarcoding methods on 90 faecal samples of snow leopards collected in Kyrgyzstan, employing one vertebrate and four plant markers. We found that argali (*Ovis ammon*) was detected only from male snow leopards. *Myricaraia* sp. was the most consumed among 77 plant operational taxonomic units found in snow leopard samples. It frequently appeared in samples lacking any prey animal DNA, indicating that snow leopards might have consumed this plant especially when their digestive tracts were empty. We also observed differences in the patterns of plant consumption between male and female snow leopards. Our comprehensive overview of prey and plants detected in the faeces of snow leopards and other sympatric mammals will help in formulating hypotheses and guiding future research to understand the adaptive significance of plant-eating behaviour in felids. This knowledge supports the enhancement of their captive environments and the conservation planning of their natural habitats.

## 1. Introduction

Animals interact with plants in multi-faceted ways, using them as sources of food, shelter, tools and medicine. When studying diets, researchers have predominantly focussed on plant species as primary food sources for herbivores and omnivores. Plants are not easy to digest, especially due to their cellulose-rich cell walls [1,2]. The inherent structural distinctions between plant and animal cells, compounded by potential toxicities in some plant species, necessitate specialized digestive and detoxification mechanisms for those consuming a predominantly plant-based diet [3–5].

Felids are primarily recognized as obligate carnivores. They possess specific morphological, physiological and behavioural adaptations that enable them to efficiently consume other animals. These adaptations, including specialized dentition for slicing and a shorter digestive tract, reflect their carnivorous diet [6–8]. The shorter digestive tract is an adaptation to their reduced need for fermentation [9], as animal tissue is easier to digest than plant matter [10]. Furthermore, felid taste receptors have evolved in response to their dietary needs, showing decreased sensitivity to fruit sugars but increased sensitivity to amino acids and lower tolerance to bitter compounds [11–16]. Although their morphological and physiological states are highly tuned to carnivorous diet, carnivores have been observed consuming plants, both in the wild and in captivity [17–19]. Plant occurrence in faeces was reported in 24 extant felid species [18], and camera traps captured three wild felid species eating grass in Costa Rica [17].

The reasons and implications behind interactions between felids and plants remain ambiguous. Plant-eating behaviour might be overlooked due to the presumption that plants play a minimal role in felid nutrition or might be ingested inadvertently (e.g. [20,21]). Considering captive felids thrive without consuming plant materials in zoos, little is known about their necessity for survival. Some posit plants act as supplementary food or moisture sources, as indicated by the presence of fruit seeds in felid faecal samples [22,23]. Others suggest that plants serve medicinal purposes, aiding in parasite excretion [24,25] or digestion [26]. A commonly held notion also suggests plants assist in the evacuation of hair and undigested material [27–29].

Understanding which plants wild felids use is pivotal for experimental design and hypothesis formulation to explore adaptive significance of the behaviour. It is important to use similar plants to the ones consumed in the wild when conducting experiments related to plant-eating behaviour. Given that plant-eating behaviour is both common and natural among wild felids [18], information about the plants they consume can enhance their captive environments (e.g. introduce plant species consumed by wild individuals into enclosures) and aid in the conservation planning of their natural habitats. Additionally, it enriches our understanding of the ecological interactions between felids and plants.

The snow leopard (*Panthera uncia*) is native to the high mountains of central Asia. Plant material has been reported in the faeces of 24 out of 41 extant felid species; notably, snow leopard faeces frequently contained plant materials, despite their alpine habitat where vegetation is typically sparse [18]. Previous studies have made cursory mentions of grasses and bushes; in particular, *Myricaria* spp. [30], and 45% of their faeces contained the shrub *Myricaria* spp. [31] in prey animal surveys but have not investigated the plant species further. Therefore, it is unclear if *Myricaria* spp. is more frequently consumed than other plants and if this is a phenomenon specific to snow leopards compared to other animals. We believe that investigating the plant repertoire consumed by wild snow leopards in alpine environments will deepen our understanding of the plant-eating behaviour, including which plants they consume despite limited plant resources.

The molecular approach using the next-generation sequencing is widely used in diet analysis for many animals [32]. DNA metabarcoding employs specially designed universal primer pairs to amplify standardized regions of DNA. These regions are then sequenced and matched against reference databases for taxonomic identification [33]. When combined with next-generation sequencing, this technology enables the concurrent taxonomic analysis of thousands of samples efficiently and economically [34]. DNA metabarcoding is considered suitable for identifying the potentially diverse dietary plants in carnivores. However, application of metabarcoding method for plant identification in felids is quite limited. A study of leopard cat (*Prionailurus bengalensis*) in China is the only case at the moment [25], and there are few studies that use this method for large cat species.

The primary objective of this study was to comprehensively identify plant species in the faeces of wild snow leopards. This would enable us to infer the traits of plants they frequently consume and the function of plant-eating. Based on Illumina sequencing data, we first revealed the frequently consumed prey and plant taxa in snow leopard faeces. Additionally, we identified the dietary plant species consumed by other mammals inhabiting the same alpine ecosystem. These included ibex (*Capra sibirica*) and argali (*Ovis ammon*) that constitute the primary prey for snow leopard, wolf (*Canis lupus*) that is another apex predator species in its habitat and red fox (*Vulpes vulpes*) that functions as a mid-level predator and omnivore. By contrasting the dietary composition of the other mammal species, we can understand the characteristics of plant eating of the snow leopard. It is equally critical to consider intraspecies variations, such as differences between sexes. A study of Puma showed that sex affects the species and size of prey [35]. Additionally, the difference in reproductive roles between sexes influences their behaviour and energy requirements [36], potentially impacting plant-eating behaviour. Consequently, we tried to find out whether the dietary composition differs between sexes in snow leopards.

## 2. Material and methods

### 2.1. Ethical note

This research adhered to the legal requirements of the governments of Kyrgyzstan and Japan. All sampling procedures were non-invasive, granted by the State Agency on Environment Protection and Forestry (now Ministry of Natural Resources, Ecology and Technical Supervision) of the government of Kyrgyzstan, and carried out according to the guidelines for animal studies in the wild and ethics in animal research issued by the Wildlife Research Center of Kyoto University.

### 2.2. Study area

The Sarychat-Ertash Reserve (42°02′N 78°25′E) spans 1341 km² in the Central Tien-Shan Mountain range’s Uch-Kol River basin. It is characterized by altitudes of 2000–5500 m and experiences a cold continental climate with mean monthly temperatures in June and January of +4.2 and −21.5°C, respectively, and annual precipitation of 295 mm. The Reserve’s vegetation consists of arid grasslands, wet meadows and tundra cushion plants [37]. Snow leopard, wolf and red fox are the most common carnivores; brown bear (*Ursus arctos*), lynx (*Lynx lynx*), Palla’s cat (*Otocolobus manul*) and stone marten (*Martes foina*) are the other carnivores found there. In addition to ibex and argali, potential snow leopard and wolf prey species include marmot (*Marmota baibacina*), hare (*Lepus tolai*), pika (*Ochotona roylei*) and birds such as snowcock (*Tetraogallus himalayensis*) and chukar partridge (*Alectoris chukar*). There are reports of four species of mustelids and four vole species in the area [37]. Historically affected by human activities such as livestock grazing and illegal hunting, only a small part of the Reserve’s buffer zone is used for seasonal livestock grazing.

### 2.3. Sample collection

The faecal samples were collected in November 2017, March and September 2018, May 2019, October 2022 and May 2023. High water levels in the summer and thick snow cover in the winter prevented field-work in these seasons during the year. Faecal samples were collected opportunistically. We collected faecal droppings of ungulates in addition to those of carnivores in the autumn of 2022. Typically, whole faeces were collected into plastic bags with silica gel after photographing it in its natural setting. Geographical coordinates, altitude and sampling time were recorded. Since refrigerating facilities were not available at the study site, faecal samples were stored in a dark place at ambient temperature until they were brought to Bishkek city. Surface of each faeces was swabbed by sterile cotton swab and preserved in sterilized 2 ml plastic tubes with 1 ml lysis buffer (0.5% sodium dodecyl sulfate, 100 mM ethylenediaminetetraacetic acid (pH 8.0), 100 mM Tris–HCl (pH 8.0) and 10 mM NaCl [38]), mixed by tapping the tube, and kept in dark boxes at ambient temperature for later processing. To reduce the environmental contamination, we cut the faeces with sterile tweezers and transferred the inner parts into 2 ml sterilized plastic tubes with 1 ml RNA*later* solution (Thermo Fisher Scientific, Waltham, MA). The contents in the tube were mixed by tapping the tube, and they were kept in a dark box at ambient temperature for later processing. Samples in the lysis buffer, specifically surface swabs, were used for species and sex identification due to the expected higher concentration of host DNA. The samples taken from the inner region of faeces were used for diet analysis.

### 2.4. DNA extraction

All experimental procedures were performed under sterile conditions, as recommended by Hayakawa *et al*. [39]. DNA from each faecal sample was extracted and purified using the QIAmp DNA Fast Stool Mini Kit (Qiagen, Hilden, Germany). DNA from samples stored in the lysis buffer was extracted according to the manufacturer’s protocol. Samples stored in RNA*later* were first precipitated and then washed twice with 1 ml of phosphate-buffered saline (pH 7.4; centrifugation speed: 20 000*g* for 10 min). Each of the processed samples was disrupted using four zirconia beads (3 mm in diameter) and 1 mg zirconia/silica beads (0.1 mm in diameter) in a 2 ml plastic tube at 4200*g* for 5 min. The DNA samples were then purified using the QIAmp DNA Fast Stool Mini Kit and eluted in 100 μl of Buffer ATE with 30 min of incubation at ambient temperature. The DNA concentrations were estimated with a Qubit dsDNA HS Assay Kit and a Qubit fluorometer (Thermo Fisher Scientific). The purified DNA samples were stored at 4°C.

### 2.5. Species identification

We used molecular species identification to identify the specific origin of each faecal sample. To accomplish that we designed a 16S rRNA primer pair (16SrRNA_L2513_felid: GCCTGTTTACCAAAAACATCAC; 16SrRNA_H2714_felid: CTCCATAGGGTCTTCTCGTCTT) to amplify a –244 bp (excluding the primers) mitochondrial 16S rRNA gene sequence (table 1). The PCR conditions and programs are provided in the electronic supplementary file. The PCR products were purified by using a High Pure PCR Product Purification Kit (Roche, Basel, Switzerland). Direct sequencing was performed using the Big Dye 3.1 Terminator cycle-sequencing kit (Applied Biosystems, Foster City, CA) according to the manufacturer’s instructions. The cycle sequencing products were purified by ethanol precipitation and nucleotide sequences were determined using an ABI PRISM 3130xl genetic analyzer (Applied Biosystems). Forward and reverse complement sequences were aligned using MEGA11 [47]. The resulting sequences were searched in the GenBank nucleotide (nt) database and species identity was determined based on the matches with the highest similarity scores (95–100%).

**Table 1.** List of primers used in the study.

**u**sage	**n**ame	**p**rimer sequence (5**′′**–3**′**)	**r**eference
species identification	16SrRNA_L2513_felid	GCCTGTTTACCAAAAACATCAC	this study
	16SrRNA_H2714_felid	CTCCATAGGGTCTTCTCGTCTT	
sex identification	ZFX-PF	TACCGAGCGATATAGCTCCAG	Sugimoto *et al*. [40]
	ZFX-PR	GTGTTCCTACGTTAAGCTATTG	
	DBY7-PF	CTCATGAAGCCCTATTTTTGGTTG	
	DBY7-PR	ACGGCGTCCGTATCTTCCA	
diet analysis	12SV5F	TAGAACAGGCTCCTCTAG	Riaz *et al*. [41]
	12SV5R	TTAGATACCCCACTATGC	
	UniplantF	TGTGAATTGCARRATYCMG	Moorhouse Gann *et al*. [42]
	UniplantR	CCCGHYTGAYYTGRGGTCDC	
	rbcL-F	CTTACCAGYCTTGATCGTTACAAAGG	Erickson *et al*. [43]
	rbcL-R	GTAAAATCAAGTCCACCRCG	Kress and Erickson. [44]
	trnL-g	GGGCAATCCTGAGCCAA	Taberlet *et al*. [45]
	trnL-h	CCATTGAGTCTCTGCACCTATC	
	ITS1-F	GATATCCGTTGCCGAGAGTC	Baamrane *et al*. [46]
	ITS1Poa-R	CCGAAGGCGTCAAGGAACAC	

### 2.6. Sex identification

After identification of species as snow leopard, we identified sex of the individual that the sample belonged to. We used one set of four primer targeting introns of zinc-finger in X chromosome; ZFX-PF/PR and DEAD box polypeptide in Y chromosome; DBY7-PF/PR [40] (table 1). The PCR conditions and programs are provided in the electronic supplementary file. PCR products were electrophoresed and visualized on 2.0% agarose gels. The same procedure was repeated at least twice, and the sex was determined only when the results were consistent.

### 2.7. Library preparation and amplicon sequencing

Library preparation and amplicon sequencing were performed with the MiSeq system (Illumina, Inc., San Diego, CA) according to the manufacturer’s protocol with modifications optimized for our sample as follows. Five different marker sets were used to analyse species’ diets. A universal vertebrate 12SV5 marker [41]; three universal plant markers: Uniplant [42], rbcL mini-barcode [43,44], trnL-g/h [45]; and finally, one Poaceae-specific marker; ITSPoa [46], to increase the taxonomic resolution for grasses (table 1). These primers were fused with 3-6-mer Ns and specific overhang adapters 5′-TCGTCGGCAGCGTCAGATGTGTATAAGAGACAG-(forward primer)-3′ and 5′-GTCTCGTGGGCTCGGAGATGTGTATAAGAGACAG-(reverse primer)-3′. PCR was performed using the KAPA HiFi HotStart ReadyMix PCR Kit (Kapa Biosystems, Inc., Wilmington, MA) with 200 nM of each primer and 25 ng DNA as the template in a total volume of 25 μl. The PCR conditions and programs are in the electronic supplementary file. When 25 ng DNA was unavailable due to low DNA yield, the available maximum volume of the DNA solution was used in the PCR. The resulting amplicons were visualized on agarose gels.

Each PCR product (20 μl) including negative controls was purified using 36 μl Agencourt AMPure XP beads (Beckman Coulter, Inc., Carlsbad, CA) with 80% ethanol washes. Each of the purified PCR products was eluted in 10 mM Tris–HCl (pH 8.5). Using the KAPA HiFi HotStart ReadyMix PCR Kit and the Illumina Nextera XT Index Kit v2, specific dual indices and sequencing adapters were attached to each amplicon by PCR conducted in a 50 μl solution containing 5 μl of each of the forward and reverse primers and 5 μl of the first purified PCR solution. The resulting amplicons were visualized on agarose gels. Each product (45 μl) was purified using Agencourt AMPure XP beads with 80% ethanol washes. Each of the purified products from the second PCR was eluted in 27.5 μl of 10 mM Tris–HCl (pH 8.5).

The DNA concentration of each product was measured with a Qubit dsDNA HS Assay Kit. Products were mixed in the same amount of DNA concentrations to form the pooled sequencing library. Fragment size distribution of the library was estimated with an Agilent 2200 TapeStation (Agilent Technologies, Inc., La Jolla, CA, USA). The library was diluted to 15 pM and subjected to a sequencing run mixed with other libraries unrelated to this study and 30% PhiX spike-in on an Illumina MiSeq sequencing platform using the MiSeq Reagent Kit v3 (600 cycles). Sequencing was separately operated in four different runs. The read lengths from the MiSeq run were 301 bp (forward sequences), 8 bp (forward indices), 8 bp (reverse indices) and 301 bp (reverse sequences). Although quality scores of nucleotides at the 3′-end of Illumina sequences are generally low, the amplicon sizes of this study were smaller than the number of cycles of the kit (i.e. 600). Therefore, overlapping regions of the forward and reverse reads were used to restore these low-quality sequences in the following bioinformatics procedure.

### 2.8. Bioinformatics

As suggested in [48], we converted the raw MiSeq BCL data into FASTQ data by ourselves using the bcl2fastq v. 2.20.422 program distributed by Illumina to prevent the potential de-multiplexing errors, and we then de-multiplexed the FASTQ data using the program Claident v. 0.9.2022.04.28. In the de-multiplexing and primer-trimming process with Claident, all the sequencing reads containing low-quality index (quality scores <30) sequences were eliminated and no mismatch between input and output index sequences was tolerated. Adapter sequences were trimmed using Skewer; https://sourceforge.net/projects/skewer [49], and the forward and reverse sequences were corrected with DADA2; https://github.com/benjjneb/dada2 [50] package on R programming interface [51]. Reads containing ambiguous bases were removed and trimming lengths were adjusted based on sequence quality profiles, so that Q-scores remained above 30. Error model calculation (for R1F/R2R read pairs and then R2F/ R1R read pairs), read correction, read merging and chimaera removal was performed at default settings implemented in DADA2. All the resulting amplicon sequencing variant (ASV) tables were curated with LULU [52] package on R to remove spurious ASVs. As the aim of the present study was to detect and identify species, and not intraspecific variation, we decided to create clusters of sequences, instead of denoising and creating ASV [53,54]. According to the developers of LULU algorithm, incorporation of DADA2 and LULU is a safe pathway for producing reliable and accurate metabarcoding data [52]. The LULU curation requires an external algorithm to produce the match list. Thus, we used VSEARCH v. 2.21.1 as recommended by the developers [52].

The remaining operational taxonomic units (OTUs) were then subjected to molecular taxonomic identification based on the automatic database search algorithm of the query-centric auto-*k*-nearest-neighbor (QCauto) method [55] and subsequent taxonomic assignment with the lowest common ancestor (LCA) algorithm [56] using Claident. Among the filtered databases bundled with Claident, we used the ‘animals_mt_genus’ and ‘animals_mt_species’ sub-databases for 12SV5 region; ‘plants_rbcL_genus’ and ‘plants_rbcL_species’ sub-databases for rbcL mini-barcode; ‘plants_cp_genus’ and ‘plants_cp_species’ sub-databases for trnL g-h; ‘overall_genus’; ‘overall_species’ databases for Uniplant and ITS1Poa. The QCauto search information was then subjected to taxonomic assignment with the LCA algorithm (LCA/genus results). As the default setting of the LCA algorithm sometimes returns conservative results, additional taxonomic assignment was conducted with a relaxed setting tolerating 5% mismatches of taxonomic information among database sequences in the LCA process; relaxed-LCA/genus [55]. The overall identification results were obtained by merging the LCA/species, LCA/genus and relaxed-LCA/genus results in this priority order using the ‘clmergeassign’ command of Claident. Since the QCauto method is conservative [55], we conducted additional megablast search for 12SV5 marker and complemented the taxonomic assignment. If an OTU was assigned to several species and we knew which candidate species inhabited the study area [57], we assigned the inhabiting species to the OTU. When several local species were assigned with same probability or no species was assigned with >95% match, we kept the QCauto result.

Index hopping rate of MiSeq is estimated to be 0.001 [58]. To ensure that index hopping did not result in false positives, the reads of the OTU in the samples were removed whenever the number of reads of an OTU detected in each sample were <0.001 of the number of reads of the OTU detected in all samples [59]. A recent study showed that a combination of a sample-based threshold with removal of maximum taxon contamination is an optimal method to remove artefacts [60]. Following the suggested filtering process [60], read counts within a sample that are less than a proportion of the total sample read count for that sample were removed. We decided the threshold proportion to 0.01 and 0.05 for the universal markers [43,61,62] and ITS1Poa [63], respectively. Threshold proportion of 12SV5 varied from 0.001 [64] to 0.05 [65], thus we chose 0.01 as other universal markers. In addition, we removed any read count within each OTU that lower than the highest read count within a negative control or blank cells for that OTU [60]. Based on the molecular taxonomic identification results, non-target OTUs (non-vertebrate and human in 12SV5, non-plant for the three universal plant markers, and those not in Poaceae family for ITS1Poa) were excluded. The OTUs from host carnivore species were also excluded from the 12SV5 dataset. The sequencing read set of each sample was rarefied to the minimum coverage rate among the analysed samples [66] using vegan [67] package of R. The coverage rate of each marker was 1.00, 1.00, 0.83, 0.83 and 1.00, for 12SV5, Uniplant, rbcL, trnL and ITS1Poa, respectively.

In order to overcome problems of primer specificity and bias, we integrated information from the four molecular markers used for plant identification using the Python 3.0 script [62]. The script provides a single list of taxa detected per sample controlling for duplications by collapsing less resolved taxa detected by one marker with higher resolved taxa detected using a different marker [62]. The ITS1Poa marker was Poaceae-specific marker to improve the resolution of grasses. Considering that Poaceae are common and faecal samples are often on the ground with grasses, Poaceae-specific amplification may increase the risk of amplification of rare sequences contaminated from the environment. Therefore, data from the ITS1Poa marker were only merged to the samples in which Poaceae sequence was detected by other three universal makers.

### 2.9. Statistical analysis

Visualization and basic statistical analyses were performed using the phyloseq v.1.26.1 [68] package in R. Dietary data were summarized across samples using two occurrence-based metrics commonly used in molecular dietary data analysis: (i) frequency of occurrence (FOO) and (ii) weighted per cent of occurrence (wPOO) [69]. The number of samples that contain a given food item is expressed as FOO, whereas wPOO weighs each occurrence according to the number of food items in the sample (i.e. lower weights to individual food taxa in a mixed meal), which is considered to be more biologically realistic [69]. Since we merged data from multiple markers, we did not use a sequence abundance-based metric. The samples without any prey or plant OTUs were excluded from subsequent statistical analyses.

A machine learning-based classification approach was applied to clarify the difference in dietary plant composition of snow leopards and other mammals. We used machine learning models using the randomForest package [70] in R to determine which plant genera best discriminated whether a sample came from snow leopard or other sympatric mammals based on the sample–plant matrix [71,72]. RandomForest evaluates an ensemble of decision trees to perform classification [70], in this instance, it classifies snow leopards and other mammals based on the plant composition in their faecal samples. Random forest models are considered to be robust against overfitting and known to have high predictive accuracy [73]. We tuned the random forest models to determine the number of variables (mtry) to try at each node of the tree that resulted in the lowest out-of-bag (OOB) error rates using randomForest function. OOB error is an internal validation method, estimating the prediction error of random forest models by using bootstrap samples not included in the construction of each tree. Since the number of samples were biased toward snow leopard, classwt option with inverse of the ratio of the sample size was used to enforce penalties for errors in minority category. Random forests estimate the variable importance. Thus, we were able to identify which plant genera represents the snow leopard faeces. Random forests provide two indicators for variable importance: mean decrease accuracy (MDA) and mean decrease gini (MDG). MDG is considered to be more stable than MDA [74], therefore we used MDG as the indicator.

A post hoc probabilistic co-occurrence analysis was conducted to show which taxa are simultaneously present in the same faecal samples of predators using package co-occur [75] in R. A prey-specific co-occurrence would indicate secondary predation of prey gut content [76]. In addition, we summed up all prey OTUs as single ‘prey’ OTU and evaluated co-occurrence with each plant OTU that indicated accidental intake from the environment such as grasses on the ground. Conversely, when a plant OTU negatively co-occurred with prey OTUs, it is more likely to be consumed by snow leopards.

The difference in the dietary composition between each sex of snow leopard was assessed by permutational analysis of variance (PERMANOVA) using the adonis2 function with 9999 permutations and visualized by non-metric multi-dimensional scaling (NMDS) using Bray–Curtis dissimilarity, as implemented in vegan [67].

Constrained analysis of principal (CAP) coordinates [77] was performed to evaluate dietary composition differences between male and female snow leopards (model 1:dietary animal composition, model 2: dietary plant composition) while accounting for the effects of sampling season and spatial autocorrelation. Spatial autocorrelation variables were added to consider the effect of spatial proximity on the sample. We first generated a set of Moran’s eigenvectors from the coordinates of each sampling point using distance-based Moran’s eigenvector maps; MEMs [78]. We then identified positive MEMs that significantly (*p* < 0.05) described spatial patterns using the function ‘moranNP.randtest’ using the R package adespatial [79]. The two CAP models were constructed by setting the Bray–Curtis dissimilarity as a response variable. The models included ‘Sex’, ‘Sampling season’, ‘Altitude’ and MEM vectors as explanatory variables. April and May were defined as spring while September and October were defined as autumn. The variables’ variance inflation factors (VIFs) were computed to check the collinearity. If the variable’s VIF was above 20, the variable was excluded from the model [80], resulting in different degrees of freedom in MEMs. The significance of models and explanatory variables were tested using permutational analysis of variance with 9999 permutations.

## 3. Results

### 3.1. Summary of sequence data

We collected 150 mammal faecal samples in total out of which, we could genetically identify the host species of 126 samples. These samples (90 snow leopards; 7 wolves; 9 red foxes; 3 brown bears; 9 ibexes; 7 argali; 1 marmot) were used in the dietary analysis. We obtained 16 623 180 raw sequence reads after de-multiplexing (27 567 reads per sample on average; standard deviation 25 858 reads). The 11 453 323 (20 862 reads per sample on average; standard deviation 21 185 reads) that passed the filtering processes were used as curated OTUs in the following analysis. In total, 13 prey animal OTUs were identified, with 11 at the species level, 1 at the family level and 1 at the order level. Additionally, of the 141 plant OTUs, 7 were identified at the species level, 81 at the genus level, 49 at the family level and 1 at the order level.

The results from FOO and wPOO were qualitatively similar, thus we show wPOO-based results for subsequent dietary analysis in the main text. The results from FOO were shown in the electronic supplementary file, figures S1–S4.

For snow leopard samples, on average, 21 367 reads per sample passed the filtering processes for animal, and 15 319 reads per sample passed for plants. We detected 8 animal prey OTUs and 77 plant OTUs. There were 51 samples from male and 27 samples from female snow leopards. We could not determine the sex in the remaining 12 samples.

### 3.2. Prey

**Figure 1.**
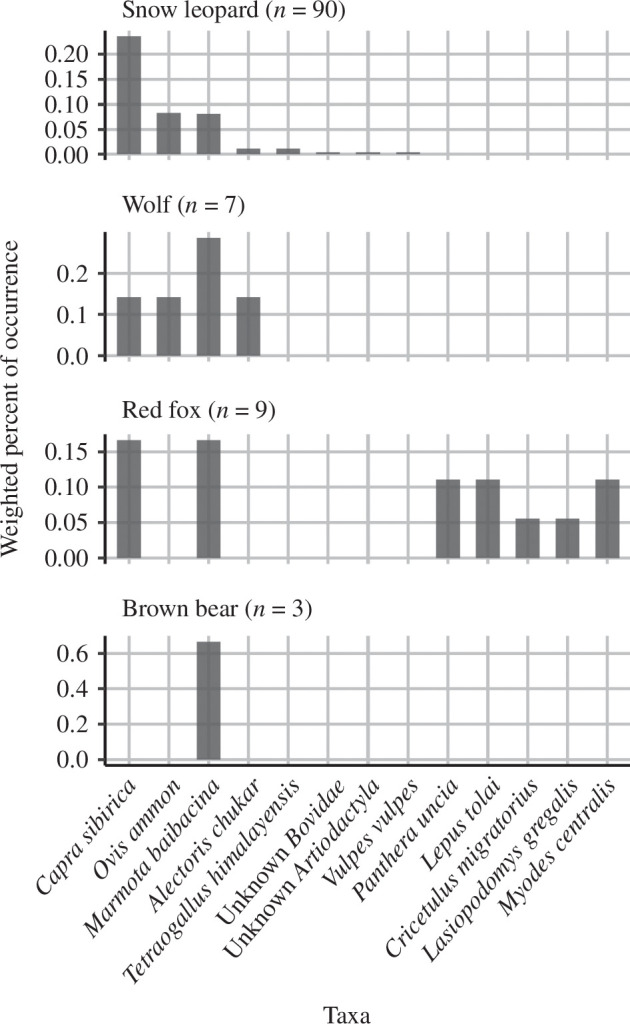
Weighted per cent of occurrence of vertebrate taxa for predators. The number in the parentheses shows the number of faecal samples.

In total, 13 OTUs were found from predator samples (table 2). Wild ungulates and marmots were the frequently detected prey species in Sarychat-Ertash for the larger predators. Ibex was the main prey of snow leopards, whereas wolves and red foxes preyed more on marmots (figure 1, electronic supplementary material, figure S5). We detected smaller mammals mainly in red fox faecal samples.

**Table 2.** The number of identified taxa for each marker. The total number of plant OTUs after merging the four plant markers is labelled as ‘merged’.

**m**arker	**o**rder	**f**amily	**g**enus	**s**pecies	OTUs
12SV5	5	7	11	11	13
uniplant	11	17	25	5	69
rbcL	9	13	13	1	34
trnL	20	28	19	0	62
ITS1Poa	1	1	10	1	48
merged	21	29	44	7	141

**Figure 2.**
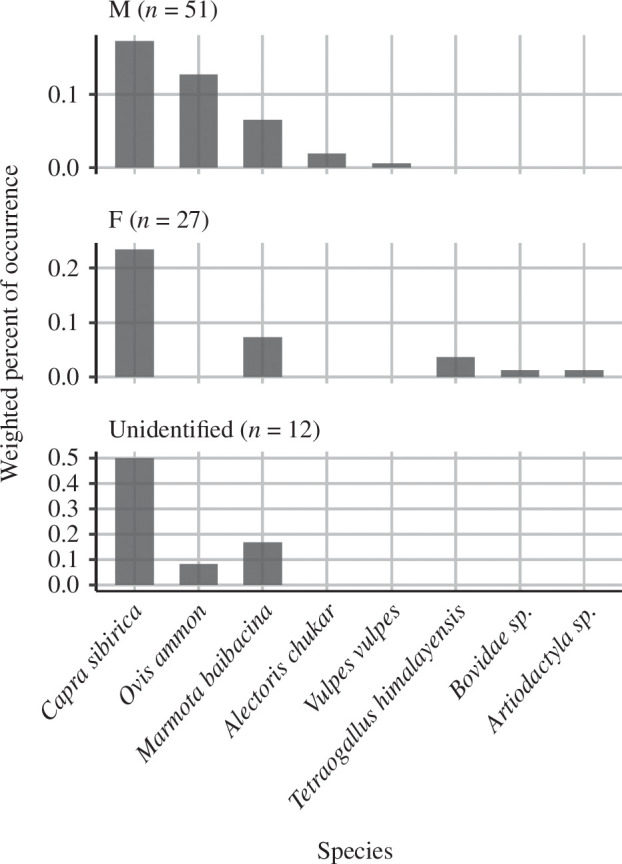
Weighted per cent of occurrence of vertebrate taxa in snow leopard faeces. The caption ‘M’ represents male samples and ‘F’ represents female samples. The number in the parentheses shows the number of faecal samples.

Remarkably, we found no OTU of argali in samples from female snow leopards (figure 2). However, the result of PERMANOVA (*p* = 0.128) as well as CAP model (*p* = 0.576) did not show a significant difference between male and female for prey composition (table 3). The CAP model controlled the effect of spatial proximity and sampling season and explained 85% of total variance.

**Table 3.** CAP coordinates for factors structuring the prey and plant composition in snow leopard samples. Models included sex, altitude, sampling season and the MEM vectors (i.e. horizontal spatial structure) as explanatory variables. Model 1 included wPOO-based OTU matrix of prey and model 2 included that of plant. Significant variables are highlighted in bold.

**m**odel	OTU matrix	**e**xplanatory variable	d.f.	***F***	***p***
model 1	prey	sex	1	0.7	0.576
		altitude	1	1.4	0.272
		season	1	1.7	0.197
		**MEMs**	18	2.1	0.019
model 2	plant	**sex**	1	2.7	0.008
		altitude	1	0.3	0.984
		season	1	1.6	0.099
		**MEMs**	21	1.4	0.003

### 3.3. Plant

**Figure 3.**
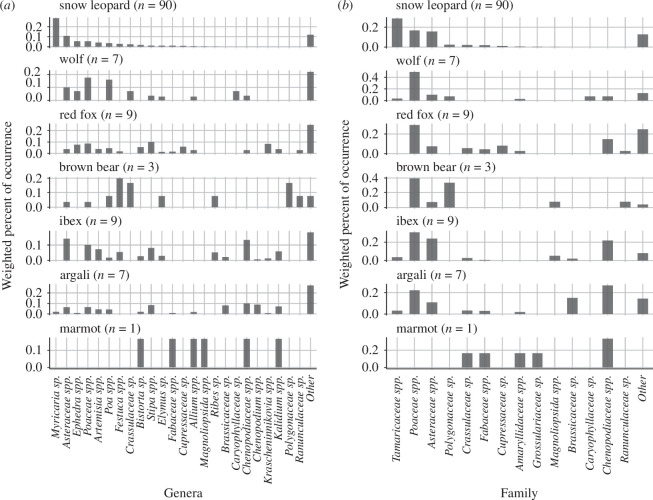
Weighted per cent of occurrence of (a) the five most frequent plant genera and (*b*) the three most frequent plant families in faeces from each mammal. Less-frequent taxa were summarized as ‘other’. The number in the parentheses shows the number of faecal samples.

The Uniplant, rbcL, trnL and ITS1Poa markers detected 69, 34, 62 and 48 OTUs, respectively (table 2). The composition of detected plant taxa was similar among Uniplant and rbcL, but trnL showed different compositions (electronic supplementary material, figures S6–S9). The merged OTU table contained 141 OTUs. The order Caryophyllales was the most common in snow leopard samples from Sarychat-Ertash Reserve. Three plant families, Asteraceae, Tamaricaceae (*Myricaria* sp.) and Poaceae showed high wPOO (0.16, 0.29 and 0.17, respectively) in snow leopard samples (figure 3). While Asteraceae and Poaceae were also detected from other mammals, Tamaricaceae was rarely detected in other species. Wolf and fox samples often contained Poaceae (wPOO: 0.49 and 0.29, respectively), whereas argali and ibex typically consumed Asteraceae (wPOO: 0.11 and 0.24, respectively), Poaceae (wPOO: 0.22 and 0.31, respectively) and Chenopodiaceae; *Lepidium* spp., *Chenopodium* spp. and *Krascheninnikovia* spp. (wPOO: 0.27 and 0.22, respectively) in autumn.

We achieved a final OOB error rate of 12.61%. The model correctly identified snow leopards in 78% of the samples it labelled as snow leopards (precision), and it correctly found 83% of the actual snow leopard samples in the dataset (recall). *Myricaria* sp. was notably important plant genera to distinguish snow leopard samples from other sympatric mammals with MDG value of 13.3, compared with 3.9 for the next most important genus, and wPOO of this genus was higher in the snow leopard samples (electronic supplementary material, figure S10).

**Figure 4.**
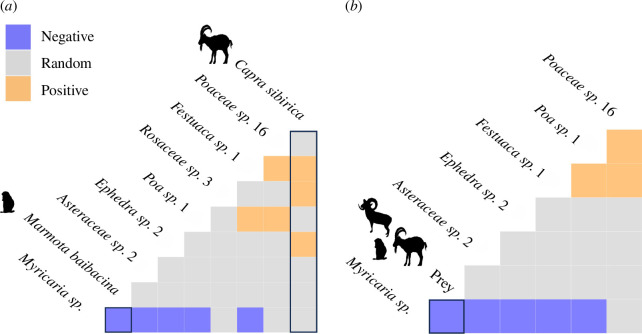
Co-occurrence matrix of plant OTUs and (*a*) each prey OTUs, (***b***) summarized prey OTU. Names of OTUs are positioned to indicate the columns and rows that represent their pairwise relationships with other OTUs. The colour of each cell represents positive, negative and random co-occurrence. Cells that show positive and negative co-occurrence of prey and plant were highlighted.

The post hoc co-occurrence analysis showed that *Myricaria* sp., which was a representative plant genus in snow leopard faeces, was negatively co-occurring with prey DNA; *p* < 0.001 (electronic supplementary material, figure S11) while Festuca, Rosaceae and *Ephedra* spp. OTUs in snow leopard faeces co-occurred with ibex OTU; *p* = 0.002, 0.006 and 0.006, respectively (figure 4).

**Figure 5.**
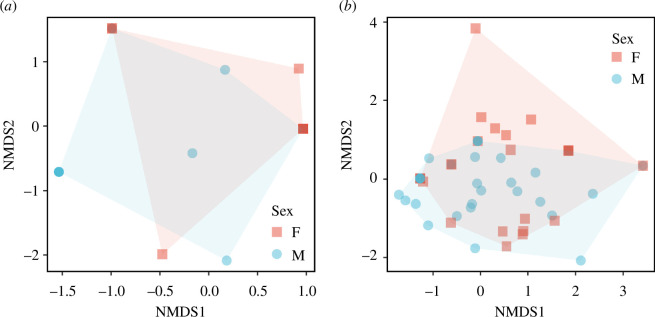
NMDS plot of wPOO-based Bray–Curtis dissimilarity of (*a*) vertebrates from snow leopard samples (stress: 0.138) and (*b*) plants from snow leopard samples (stress: 0.067). Colour and shape correspond to different sex.

Figure 5*b* shows the NMDS plot of plant composition (stress: 0.067) where we removed outliers to make it easy to interpret. The CAP model explained 49% of the total variances and showed that plant composition was different among sex (*p* = 0.008), as well as the result from PERMANOVA (*p* = 0.049). The effect of the sampling season (spring or autumn) was suggested, though it was not statistically significant (table 3). *Myricaria* sp. was not detected from female samples in autumn, when the presence of *Ephedra* spp., Asteraceae, Poaceae and Crassulaceae increased. In case of males, *Myricaria* sp. was detected during both seasons.

## 4. Discussion

### 4.1. Prey

Carnivores in Sarychat-Ertash relied on wild ungulates and marmots as reported in previous microhistological research [31]. Conducted between June and October in 2009 at the same study site as ours, Jumabay-Uulu [31] reported higher occurrence of argali than ibex in the diets of snow leopard and wolves (18:3 for snow leopards and 12:8 for wolves). In contrast, our study finds occurrence of argali to be lower than that of ibex in the carnivore diets [31]. This variation may be due to differences in methodology (e.g. microhistological versus molecular, sampling season, sampling location) or ecological factors (e.g. changes in relative abundance). Many argali died due to unexpected heavy snowfall in 2022 (Zhumabai-uulu, pers. comm.), which might have influenced the proportion of argali and ibex in carnivores' diets. One snow leopard sample contained a small number of red fox DNA reads ([red fox]:[snow leopard] = 180:8468), and two red fox samples contained a small number of snow leopard DNA reads ([red fox]:[snow leopard] = 23 892:1194 and 26 974:703). Red fox sometimes scavenges from snow leopard kill, thus snow leopard DNA in red fox samples was probably a by-product of scavenging. Red fox sometimes defecate close to the scrapings of snow leopard [81] and snow leopard is known to kill smaller predators such as red fox to prevent scavenging [82].

We did not find a significant difference in dietary prey items between sexes in snow leopard samples. One female sample contained *T. himalayensis* and another contained unidentified Artiodactyla (the order to which ungulates belong). Unidentified Artiodactyla OTU was believed to be a by-product of DNA degradation since the sample contained *C. sibirica* OTU as well (figure 5*a*). The limited diversity of potential prey mammals in the study area might have obscured any sex differences. Notably, only male samples contained traces of argali (figure 2). Although we did not identify individuals, argali was detected from male samples collected in 2018, 2022 and 2023. Females (~43 kg), being slightly smaller than males (~52 kg) might prey on argali; 60–185 kg, less often due to its larger size compared to ibex; 30–100 kg [30,83]. It will require further detailed investigation to determine whether this difference between male and female snow leopard’s consumption of argali was an artefact of the size difference between male and female snow leopards [30,83], or the possibility that with their larger home ranges [84], male snow leopards were more likely to venture into sub-optimal habitat such as rolling terrain that are used by argali [85].

### 4.2. Plant

We found various plant taxa from snow leopard samples. The frequent detection of the genus *Myricaria* agreed with previous observation-based reports in the same study sites [31]. We evaluated the potential for secondary consumption by integrating co-occurrence analysis with the examination of dietary plant items present in the faecal samples of prey. The *Festuca*, Rosaceae and *Ephedra* OTUs in snow leopard faeces were non-randomly detected with ibex OTU indicating the possibility of secondary consumption from ibex gut content. Although *Ephedra* spp. was not detected from ibex samples collected in this study, a previous study reported that livestock ate *Ephedra*’s young shoot in early spring in China [86]. In this study, ibex samples were collected in autumn, thus seasonal food plant fluctuation might have prevented the detection of *Ephedra* OTUs. It is also reported that Siberian ibex in eastern Tianshan, China preferred eating forbes (Asteraceae, Gentianaceae, Rosaceae, Fabaceae) that have higher nutritional value than graminoids during the warm season and graminoids occupied a high proportion of their diet during the cold season [87]. Since our co-occurrence analysis is only exploratory, it does not necessarily confirm ecological interactions [76]. However, given that plants that positively co-occurred were reported to be frequently consumed by ungulates, their presence in snow leopard faeces is more likely attributable to secondary consumption rather than voluntary intake by the snow leopards. Despite the lack of significant co-occurrence with prey OTUs, the frequent detection of Asteraceae in prey faecal samples indicates a potential for secondary consumption.

On the other hand, *Myricaria* spp., the representative plant in snow leopards, tended to be detected from samples which did not contain any prey OTUs. This suggests that snow leopards intentionally consumed this bush more frequently, particularly when their digestive tracts were empty. The factors that cause snow leopards to intake *Myricaria* spp. may have some relationship to whether the individual obtained prey or not. In domestic cats, it has been hypothesized that constant availability of food (ad libitum feeding) may reduce the inclination to ingest alternative items such as plastic [88]. When the digestive tracts of felids are empty, they may exhibit a tendency to bite hard objects as a means to compensate for their appetite. One species in this genus, *Myricaria bracteata*, has been used in traditional Tibetan medicine and contains anti-inflammatory compounds [89], although its medicinal effects have not been specifically tested on snow leopards. Therefore, intake of *Myricariaia* spp. and the failure to acquire prey may be related to the individual health condition of the snow leopards.

**Figure 6.**
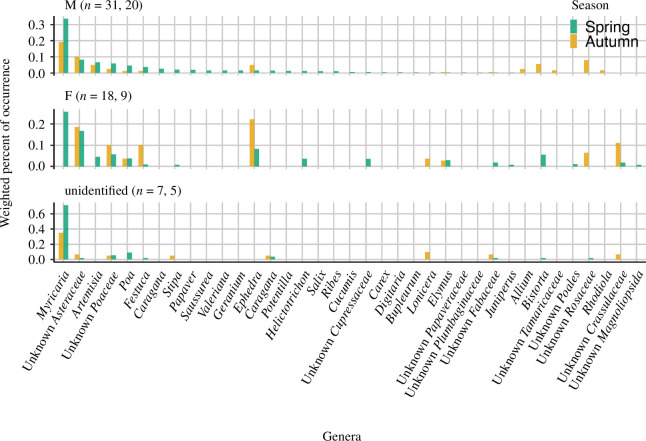
Weighted per cent of occurrence of plants from snow leopard samples. The caption ‘M’ represents males and ‘F’ represents females. The numbers in the parentheses are the number of samples collected in spring and autumn, respectively. The colour corresponds to different sampling seasons.

Female samples tended to contain *Ephedra* spp. and Asteraceae as often as *Myricaria* spp. Besides, no *Myricaria* spp. were detected from female samples collected in autumn (figure 6). As previously mentioned, *Ephedra* spp. and Asteraceae were suspected to be instances of secondary consumption. Since snow leopards give birth in mainly early summer [90], this difference could be resulting from the seasonal behavioural differences among males and females. The genus *Myricaria* is not a dominant plant in the study area and is sporadically distributed along rivers. Although there is little information about snow leopard nursing behaviour in the wild, during nursing period, mother may have preferred to stay closer to the cubs than proactively look for the *Myricaria* spp. patch. A female captive snow leopard exhibited a lower frequency of plant-eating behaviour in the year she shared an enclosure with her cub, as compared to the following year when the cub became independent [19]. While our results are indicative, it is important to consider the caveat of small sample size especially in case of female faeces. The CAP model explained only half of the total variance, thus there is a possibility of other factors, not included in this study, affecting the presence of plants in snow leopard diet.

An inter-regional sampling with a specific study design is required to better understand the relationship between snow leopards and plants. *Myricaria* spp. was often detected in snow leopard diet from other countries such as Nepal and India, but frequent containment of feather grass was reported in Mongolia [30]. Comparison of plant repertoire in different regions will provide answers to why snow leopards selectively intake on *Myricaria* spp. in this study area and identifying commonalities will lead to understanding the adaptive significance of plant-eating. In addition, a comprehensive vegetation survey is necessary to evaluate the preference in light of availability. Furthermore, adopting multi-faceted approaches, such as investigating the characteristics of the behaviour and the effects on immunity and digestion, is crucial to unravel the reasons behind plant-eating behaviour.

### 4.3. Limitation

Due to challenging terrain that limited human access, we could not establish a clear transect for sampling, and we did not identify individual animals for each faecal sample, possibly leading to sampling bias. Seasonal constraints further limited our study; for example, high water levels and deep snow prevented sampling in summer and winter. Additionally, the number of faecal samples from species other than snow leopards was limited, restricting our ability to perform statistical comparisons between species. While metabarcoding is powerful for diet analysis, its high sensitivity can also pick up environmental contamination or accidental intake [76]. We refrained from using host blocking primers to prevent unexpected amplification bias towards prey animals, though this approach might have lowered the sensitivity in detecting prey species. Sometimes, there was amplification bias as we found in plant markers in this study (electronic supplementary material, figures S6–S8). Although we took steps to minimize these biases, they couldn't be entirely eliminated. The resolution of the markers was not enough to identify plant OTUs at the species level, and incompletion of plant references due to lack of a comprehensive vegetation survey in the area could have led to inflated diversity estimates for plant OTUs.

## 5. Conclusion

In this study, we applied a molecular-based approach to comprehensively investigate animal and plant in faeces of mammals in the alpine habitat of Kyrgyzstan. Detected prey items from large carnivores agreed with previous studies in the same study site. Red fox, a mesocarnivore, consumed smaller mammals as well. Although statistical significance was not detected, consumption of argali was biased toward male snow leopards indicating the possibility of prey selection according to the predator’s body size.

We focused on dietary plants and highlighted the feature of plant repertoire in snow leopard faeces. As mentioned in an observation-based report, the genus *Myricaria* characterized the snow leopard samples. We found the plant was negatively co-occurred with animal prey DNA, indicating the consumption of this bush when the digestive tracts were empty. This suggests the importance of simultaneous investigation of prey and plant in carnivore diet.

Unveiling the relationship between snow leopards and plants, obligate carnivores and plants in general, improves our understanding of not only their behaviour and ecology but also evolution of diet repertoire and animal–plant interaction in the ecosystems. Practically, it aids in the conservation planning of felids, as conserving species interactions is considered important in conjunction with the conservation of individual species [91].

## Data Availability

The raw sequencing data and related metadata have been deposited in the DNA Data Bank of Japan database (BioProject PRJDB16690) [92]. Processed data and R code have been deposited in the Dryad Digital Repository at [93]. Electronic supplementary material is available online at [94].
